# Alkaline phosphatase activity after cardiothoracic surgery in infants and correlation with post-operative support and inflammation: a prospective cohort study

**DOI:** 10.1186/cc11483

**Published:** 2012-08-20

**Authors:** Jesse Davidson, Suhong Tong, Amanda Hauck, D Scott Lawson, James Jaggers, Jon Kaufman, Eduardo da Cruz

**Affiliations:** 1 The Heart Institute, Department of Pediatrics, Children's Hospital Colorado, 13123 East 16th Ave, Box 100 Aurora, CO 80045, USA; 2 Department of Biostatistics, University of Colorado Denver, 13001 E 17th Place, Campus Box B119, Aurora, CO 80045, USA

## Abstract

**Introduction:**

Limited evidence suggests that serum alkaline phosphatase activity may decrease after cardiac surgery in adults and children. The importance of this finding is not known. Recent studies, however, have identified a potential role for alkaline phosphatase as modulator of inflammation in multiple settings, including during adult cardiopulmonary bypass. We sought to describe the change in alkaline phosphatase activity after cardiothoracic surgery in infants and to assess for a correlation with intensity and duration of post-operative support, markers of inflammation, and short-term clinical outcomes.

**Methods:**

Sub-analysis of a prospective observational study on the kinetics of procalcitonin in 70 infants (≤90 days old) undergoing cardiothoracic surgery. Subjects were grouped based on the use of cardiopulmonary bypass and delayed sternal closure. Alkaline phosphatase, procalcitonin, and C-reactive protein (CRP) levels were obtained pre-operation and on post-operative day 1. Mean change in alkaline phosphatase activity was determined in each surgical group. Generalized linear modeling and logistic regression were employed to assess for associations between post-operative alkaline phosphatase activity and post-operative support, inflammation, and short term outcomes. Primary endpoints were vasoactive-inotropic score at 24 hours and length of intubation. Secondary endpoints included procalcitonin/CRP levels on post-operative day 1, length of hospital stay, and cardiac arrest or death.

**Results:**

Mean decrease in alkaline phosphatase was 30 U/L (p = 0.01) in the non-bypass group, 114 U/L (p<0.0001) in the bypass group, and 94 U/L (p<0.0001) in the delayed sternal closure group. On multivariate analysis, each 10 U/L decrease in alkaline phosphatase activity on post-operative day 1 was independently associated with an increase in vasoactive-inotropic score by 0.7 (p<0.0001), intubation time by 6% (p<0.05), hospital stay by 5% (p<0.05), and procalcitonin by 14% (P<0.01), with a trend towards increased odds of cardiac arrest or death (OR 1.3; p = 0.06). Post-operative alkaline phosphatase activity was not associated with CRP (p = 0.7).

**Conclusions:**

Alkaline phosphatase activity decreases after cardiothoracic surgery in infants. Low post-operative alkaline phosphatase activity is independently associated with increased procalcitonin, increased vasoactive/inotropic support, prolonged intubation time, and prolonged hospital stay. Alkaline phosphatase may serve as a biomarker and potential modulator of post-operative support and inflammation following cardiothoracic surgery in infants.

## Introduction

Alkaline phosphatase (AP) is an endogenous metalloenzyme with four distinct isoenzymes (tissue non-specific, intestinal, placental, and germ cell). Post-translational modifications of the non-specific tissue-form result in a total of six distinct liver and bone isoforms [1-3]. AP is uniformly present in serum where the primary isoforms originate from liver and bone, and to a smaller extent from the intestine.

Recently, AP has garnered renewed research interest as a component of the host defense against inflammation, in part due to its ability to neutralize endotoxin [4-9]. Exogenously administered AP produces beneficial effects on inflammation in models of ulcerative colitis [7,10], necrotizing enterocolitis [11], and sepsis [6,12-14]. Systemic inflammation is common after cardiac surgery, and profound or prolonged inflammation can adversely affect surgical outcomes [15]. Cardiac surgery and cardiopulmonary bypass (CPB) trigger the systemic inflammatory response through various mechanisms including surgical trauma, hypothermia, exposure to foreign materials and blood products, ischemia reperfusion, and splanchnic hypoperfusion with intestinal mucosal damage and endotoxin leak [15-17]. AP may have a protective role against systemic inflammation in patients after CPB [8,9].

Two prior studies, one in children undergoing repair of congenital heart disease and one in adults undergoing coronary artery bypass grafting, described significant decreases in AP activity following cardiac surgery with CPB [18,19]. Neither study was able to identify an etiology for the decrease, nor did the authors explore the clinical implications of this decrease. We sought to better describe the decrease in AP activity after cardiothoracic surgery in infants and to assess whether CPB was a necessary mediator. We further hypothesized that lower post-operative AP activity would correlate with increased inflammation, increased post-operative support, and prolonged post-operative care.

## Materials and methods

Patients were enrolled as part of a prospective, observational study examining the post-operative kinetics of the inflammatory marker procalcitonin (PCT) in infants ≤ 90 days of age, undergoing cardiothoracic surgery. The protocol was approved by the Colorado Multiple Institution Review Board. Exclusion criteria were estimated gestational age < 34 weeks or weight < 1,200grams at the time of surgery. The study was performed in a single tertiary care center with dedicated pediatric cardiovascular operating rooms and cardiac intensive care unit (CICU). Study size was determined by power analysis from the procalcitonin study. Investigation of AP was performed as a sub-analysis of the PCT trial. Patient enrollment occurred between July 2009 and September 2010. Surrogate informed consent was obtained from parents or legal guardians in all cases. All enrolled patients completed the study protocol.

Baseline demographic and surgical information collected on all patients included gender, ethnicity, gestational age at delivery, age and weight at the time of surgery, anatomic diagnosis, surgical procedure, Aristotle comprehensive complexity score [20,21], use of pre-operative steroids, CPB time, aortic cross-clamp time, and deep hypothermic circulatory arrest (DHCA) time. By protocol, all patients underwent pre-operative assessment of PCT, C-reactive protein (CRP), and liver function tests (LFTs), including total AP activity while under anesthesia immediately prior to the opening incision. AP activity was determined by the commercially available VITROS ALKP Slide method using the VITROS Chemistry Products Calibrator Kit 3 (Ortho Clinical Diagnostics, Rochester, New York, USA). Rate of change of p-nitrophenyl phosphate to p-nitrophenol by AP was monitored by reflectance spectrophotometry and the rate of change in reflection was converted to enzyme activity.

CPB was performed using a neonatal circuit consisting of a roller head pump (Terumo System 1, Ann Arbor, MI, USA) and a Terumo FX05 oxygenator with a blood prime. The prime routinely underwent hemofiltration using a Sorin DHFO.6 hemoconcentrator (Sorin Group USA, Inc., Arvada, CO, USA) with a polyethersulfone membrane prior to initiating bypass, allowing for partial filtration of molecules up to 65,000 Daltons. Anticoagulation was achieved prior to CPB by administering 400 units/Kg of heparin systemically to the patient. Initial target flow rate was approximately 200 ml/Kg/minute. Cardioplegia was accomplished using del Nido formula cardioplegia solution at an initial dose of 30 ml/Kg and subsequent dosing was considered after 45 to 60 minutes of aortic cross-clamp time. Conventional ultrafiltration was utilized throughout CPB. After CPB, modified ultrafiltration was employed to remove inflammatory mediators and increase hematocrit.

Patients were separated into three groups based on the following operative characteristics: 1) delayed sternal closure (DSC), 2) CPB without DSC, and 3) no CPB or DSC. Group assignment primarily determined the blood draw schedule for PCT and CRP. The decision to perform DSC was made by the attending surgeon as a precaution in patients with poor function, significant myocardial edema, or other concerns for low cardiac output. Invasive arterial and central venous pressure monitoring were performed in all cases. Inotropic and vasoactive medications were initiated at the discretion of the surgical team. Ongoing titration was directed by the intensivist and did not follow a pre-established protocol. Vasoactive-inotropic score (VIS) was calculated as per Gaies *et al*. [22] at 24, 48, and 72 hours after admission to the CICU. PCT and CRP levels were drawn on post-operative day 1 (POD1) in all patients. Patients in groups one and two also had LFTs drawn between 0400 and 0600 on POD1. Additional laboratory testing was performed as directed by the intensivist.

The primary study objective was to describe the changes in AP activity from pre-operation to POD1. Secondary goals included the assessment of associations between post-operative AP activity and post-operative support, inflammation, and short term outcomes. Primary clinical endpoints determined a priori were VIS at 24 hours after CICU admission (VIS24) and length of intubation in hours. Secondary endpoints included VIS at 48 and 72 hours after CICU admission (VIS48, VIS72), length of CICU and hospital stay, PCT on POD1, and CRP on POD1. Given the low expected mortality, a combined dichotomous poor outcome variable as previously described was utilized as a secondary outcome [22]. Poor outcome was defined as the occurrence of any one of the following events or interventions: cardiac arrest requiring chest compressions, death within 30 days or at any point prior to discharge, renal replacement therapy, or mechanical circulatory support.

Baseline characteristics were expressed as means with SD for normally distributed data and medians with quartiles for nonparametric data. Comparisons of baseline variables between groups were performed using the chi-square test for categorical data and two-sample *t*-test or Mann-Whitney *U*-test for continuous variables. Pre-operative and POD1 AP activity were compared within each surgical group utilizing the paired *t*-test.

Outcome distributions were examined prior to modeling. Highly skewed data were log transformed unless they contained negative values or excessive zeros, in which case the outcomes were dichotomized as upper 25^th ^percentile vs. lower 75^th ^percentile (VIS48 and 72). General linear modeling was employed to model outcomes that were normally distributed with either original data or following log transformation. Logistic regression was applied to model the dichotomized data. Potential covariates included surgical group, age, Aristotle comprehensive score, weight at surgery, bypass time, cross-clamp time, DHCA time, and the presence of single ventricle physiology. Spearman correlation testing between outcomes and candidate variables was first performed to identify potential covariates. Candidate variables with a *P*-value from correlation testing that was less than 0.1 were then selected for multiple regression. Further correlation testing was performed among identified covariates to identify potential confounders. Backwards stepwise regression was used to identify significant covariates with a *P*-value cutoff of 0.1. Surgical group was strongly associated with all outcomes and was kept in all models to control for clinical differences. Pre-operative AP activity and PCT/CRP levels were also kept in all appropriate models. All other potential covariates assessed did not contribute substantially to model fit and were excluded. R-square/adjusted R-square were used to evaluate percent variability due to AP in general linear models, while the receiver operator characteristic (ROC) curve and the area under the curve (AUC) were generated to evaluate the sensitivity and specificity in predicting dichotomized outcomes. Model results are reported as change per 10 U/L of AP for normally distributed outcomes, percentage change per 10 U/L of AP for nonparametric outcomes, and change in odds ratio per 10 U/L of AP for dichotomized outcomes. P-values smaller than 0.05 were considered statistically significant.

## Results

**Figure 1:**
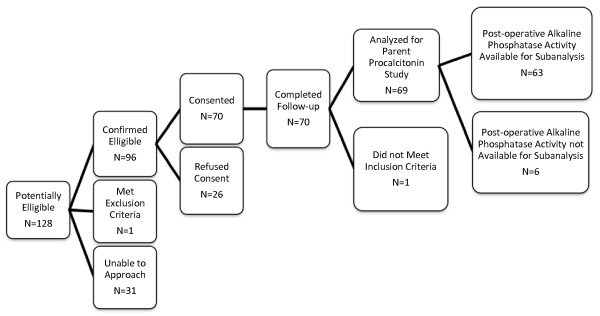
**Patient enrollment flow chart**.

Enrollment details are presented in Figure 1. A total of 70 patients were enrolled and completed the study. Of the 70 patients enrolled, 56 underwent CPB, of which 26 required DSC. One additional patient did not undergo CPB but required DSC due to shunt malfunction and immediate reoperation for shunt revision. One patient was found to have an inaccurate date of birth in the medical record, was older than inclusion criteria permitted, and was excluded from all subsequent analyses.

Demographic and baseline surgical data are shown in Table 1. Overall, the DSC and non-bypass patients were younger than the general CBP patients, and the DSC patients weighed significantly less at the time of operation. The DSC group had higher Aristotle scores, a higher prevalence of single ventricle physiology, and greater use of pre-operative steroids. Median bypass time and DHCA time were also longer in the DSC group, although aortic cross-clamp time was comparable.

**Table 1:** Baseline demographics and surgical data

	No CPB n = 13	CPB n = 29	DSC n = 27	*P*-value No CPB vs. CPB	*P*-value No CPB vs. DSC	*P*-value CPB vs. DSC
Male	8 (62%)	16 (55%)	18 (67%)	ns	ns	ns
Ethnicity				ns	ns	ns
White	9 (69%)	18 (62%)	15 (56%)			
Hispanic	4 (31%)	10 (34%)	11 (40%)			
Other	0 (0%)	1 (4%)	1 (4%)			
Age, days	11 (3, 14)	49 (9, 67)	5 (4, 19)	< 0.0001	ns	< 0.005
Weight, Kg	2.9 (2.5, 3.6)	3.4 (3.1, 4)	3.1 (2.8, 3.4)	ns	ns	<0.01
Aristotle score	8 (7, 8)	10 (7, 11)	12 (10, 15)	< 0.05	<0.0001	< 0.005
Bypass time, minutes	n/a	130 (94, 176)	167 (153, 198)	n/a	n/a	< 0.05
Cross-clamp time, minutes	n/a	86 (61, 113)	76 (56, 93)	n/a	n/a	ns
DHCA time, minutes	n/a	0 (0, 0)	22 (0, 35)	n/a	n/a	< 0.01
Single ventricle physiology	3 (23%)	2 (7%)	12 (44%)	ns	ns	< 0.005
Pre-operative steroids	1 (8%)	12 (41%)	23 (85%)	< 0.05	< 0.0001	< 0.005

Unless otherwise specified, categorical data are expressed as n (%) and continuous data are expressed as median (25^th^, 75^th ^percentile). CPB, cardiopulmonary bypass; DSC, delayed sternal closure; DHCA, deep hypothermic circulatory arrest; ns, not significant; n/a, not applicable.

Median pre-operative and POD1 AP activity are shown in Table 2. Pre-operative AP activity was significantly higher in the CPB group than in either the non-bypass or DSC groups. AP activity was assessed on POD1 in 63 of 69 patients. Two patients (one in the non-bypass group and one in the DSC group) died on POD0. Three additional patients in the non-bypass group and one patient in the DSC group did not have an AP drawn on POD1, at the discretion of the clinical team. AP activity on POD1 dropped in 62 of the 63 remaining patients compared to their pre-op activity. Median AP activity on POD1 was significantly lower in the DSC group than in the non-bypass or CPB groups. Mean changes in AP activity for patients in each surgical group are demonstrated in Table 3. All three groups showed a statistically significant mean decrease in patients' AP activity with a greater mean decrease in the CPB and DSC groups than in the non-bypass group. Changes in AP activity were not correlated with changes in albumin (*r *= 0.18, *P *not significant), total protein (*r *= 0.12, *P *not significant), or AST (*r *= -0.04, *P *not significant), but showed a moderate inverse correlation with ALT (*r *= -0.54, *P *< 0.0001).

**Table 2:** Alkaline phosphatase activity, measured pre-operatively and on post-operative day 1

	No CPB	CPB	DSC	P-value No CPB vs. CPB	P-value No CPB vs. DSC	P-value CPB vs. DSC
AP (U/L), Pre-op	143 (128, 173)	236 (150, 302)	155 (28, 297)	< 0.05	ns	< 0.005
AP (U/L), POD1	112 (70, 148)	141 (77, 166)	61 (45, 78)	ns	< 0.05	< 0.0001

Alkakine phosphatase (AP) levels are presented as median (25^th^, 75^th ^percentiles). CPB, cardiopulmonary bypass; DSC, delayed sternal closure; AP, alkaline phosphatase; POD, post-operative day; ns, not significant.

**Table 3:** Mean change in alkaline phosphatase activity measured pre-operatively and on post-operative day 1

Surgical group	Number	Mean difference, U/L (SD)	95% CI	*P*-value
No bypass	9	-29.9 (26.5)	(- 50.3, -9.5)	< 0.01
CPB	29	-114.4 (97.6)	(-152.2, -76.5)	< 0.0001
DSC	25	-93.8 (47.1)	(-112.8, -74.7)	<0.0001

CPB, cardiopulmonary bypass; DSC, delayed sternal closure; CI, confidence interval.

**Figure 2:**
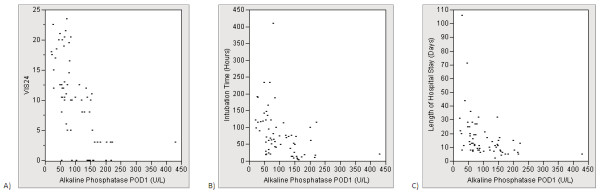
**Scatter plot for alkaline phosphatase (AP) versus VIS24 (A), intubation time (B), and length of hospital stay (C)**.

Post-operative support and short-term outcomes for each group are shown in Table 4. Overall, the DSC group required significantly more post-operative support as demonstrated by higher median VIS24, 48, and 72, and longer median intubation time, CICU stay, and hospital stay. The DSC group also exhibited a greater incidence of combined poor outcome. Spearman correlation testing was used to assess univariate associations between AP on POD 1 and post-operative support. AP activity on POD1 demonstrated a statistically significant moderate inverse correlation with VIS at 24 (*r *= -0.69; *P *< 0.0001), 48 (*r *= -0.65; *P *<0.0001), and 72 hours (*r *= -0.61; *P *< 0.0001), as well as length of intubation (*r *= -0.54; *P *< 0.0001), length of CICU stay (*r *= -0.52; *P *< 0.0001), and length of hospital stay (*r *= -0.60; *P *< 0.0001). Scatterplots displaying the relationship of AP activity to VIS24, length of intubation, and length of hospital stay across the entire cohort are shown in Figure 2.

**Table 4:** Post-operative support and outcomes

Outcome variable	No bypass n = 13	CPB n = 29	DSC n = 27	*P*-value No bypass vs. CPB	*P*-value No bypass vs. DSC	*P*-value CPB vs. DSC
Intubation, hours	28 (16, 48)	40 (18, 72)	117 (74, 165)	ns	< 0.001	<0.0001
Length of hospital stay, days	12 (6, 15)	10 (7, 14)	21 (13, 28)	ns	< 0.01	<0.0005
Length of ICU stay, days	3 (2.5, 6)	4 (3, 7)	9 (6, 15)	ns	< 0.05	<0.01
VIS24	0 (0, 3)	8 (3, 11)	15 (12, 20)	< 0.005	< 0.0001	<0.0001
VIS48	0 (0, 0)	3 (0, 7)	13 (10, 18)	< 0.05	< 0.0001	<0.0001
VIS72	0 (0, 0)	0 (0, 4)	11 (5, 15)	ns	< 0.001	<0.0001
Poor outcome	1 (9%)	1 (4%)	7 (26%)	ns	ns	<0.05

Unless otherwise specified, categorical data are expressed as n (%) and continuous data are expressed as median (25^th^, 75^th ^percentiles). CPB, cardiopulmonary bypass; DSC, delayed sternal closure; ICU, intensive care unit; VIS, vasoactive-inotropic score; ns, not significant.

We next performed general linear modeling to assess the association between AP and outcomes while controlling for potential confounding variables (Table 5). Controlling for surgical group and pre-operative AP activity, each 10 U/L decrease in AP on POD1 was independently associated with an increase in VIS24 of 0.7 (*P *< 0.0001), a 6% increase in intubation time (*P *< 0.05), and a 5% increase in length of hospital stay (*P *< 0.05). Similarly, length of CICU stay increased by 4% for each 10 U/L decrease in AP on POD1, but this increase was not statistically significant. Two additional outcomes (VIS48 and VIS72) were assessed by multiple logistic regression due to the presence of excessive zero values. Cutoffs for VIS48 and VIS72 were 12 and 10 respectively. Each 10 U/L decrease in AP was independently associated with increased odds of high VIS48 and VIS72 (*P *< 0.005). Statistics of model fit are included in Table 4. Overall, based on these models, AP activity on POD1 was responsible for between 5 and 15% of the variation in post-operative support. Additional modeling using the change in AP activity as the independent variable showed no significant association with post-operative support (data not shown).

**Table 5:** Multivariate modeling: change in outcome variable per 10 U/L decrease in alkaline phosphatase (AP) activity*

Outcome variable	AP POD1 (95% CI)	*P*-Value	*R* ^2^	AUC (95% CI)
Intubation, % change	6% (1%, 12%)	< 0.05	0.47	n/a
Length of hospital stay, % change	5% (2%, 9%)	< 0.05	0.35	n/a
Length of ICU stay, % change	4% (-3%, 8%)	ns	0.3	n/a
VIS24, unit change	0.7 (0.4, 1.0)	< 0.0001	0.70	n/a
VIS48, OR for change	1.4 (1.1, 1.7)	< 0.005	n/a	0.93 (0.87, 1.0)
VIS72, OR for change	1.4 (1.2, 1.7)	<0.005	n/a	0.89 (0.81, 0.97)
Poor outcome, OR for change	1.3 (1, 1.8)	0.061	n/a	0.78 (0.66, 0.90)
PCT, POD1, % change	14% (4%, 25%)	< 0.01	0.26	n/a
CRP, POD1, %change	-4% (-17%, 27%)	ns	0.30	n/a

*change expressed as unit change for normally distributed variables, % change for log transformed variables, and odds ratio (OR) change for data with excessive zero values (logistic regression). ICU, Intensive care unit; VIS, vasoactive-inotropic score; POD, post-operative day; PCT, procalcitonin; CRP, C-reactive protein; AUC, area under the curve; CI, confidence interval; ns, not significant; n/a, not applicable.

Poor outcome occurred in nine patients (five deaths and four additional cardiac arrests). No patients required mechanical circulatory support or renal replacement therapy. Two deaths occurred prior to assessment of AP activity on POD1 leaving a total of seven events for analysis. The association between poor outcome and AP activity on POD1 was assessed using ROC analysis. The AUC based on ROC analysis was 0.78 (95% CI 0.66 to 0.90). Of interest, all poor outcome events analyzed occurred in patients with AP activity less than 90 U/L. Multiple logistic regression modeling controlling for surgical group and pre-operative AP activity showed a trend towards increased odds of poor outcome for each 10 U/L decrease in AP activity on POD1 (Table 5).

Inflammatory biomarker data are shown in Table 6. Pre-operation, median PCT and CRP levels were normal in all groups with the exception of a mild elevation of median PCT in the non-bypass group. There was a moderate inverse correlation between pre-op PCT and AP activity (*r *= -0.47, *P *< 0.0001) and a weak inverse correlation between CRP and AP activity (*r *= -0.30; *P *< 0.05). The patients with the highest pre-operative PCT levels uniformly carried a diagnosis that placed them at risk for decreased systemic blood flow (severe left sided obstructive lesion, pulmonary atresia/intact ventricular septum, single ventricle with complete heart block) and the majority (eight out of ten) exhibited significant lactic acidosis (greater than 2 mmol/L) in the pre-operative period. On POD1, PCT and CRP levels were elevated in all surgical groups.

**Table 6:** Inflammatory markers

	No bypass	CPB	DSC	*P*-value No bypass vs. CPB	*P*-value No bypass vs. DSC	*P*-value CPB vs. DSC
PCT, ng/ml, pre-operation	0.5 (0.15, 1.16)	0.11 (0.1, 0.22)	0.2 (0.1, 0.31)	< 0.005	ns	< 0.05
PCT, ng/ml, POD1	1.1 (0.4, 2.8)	2.4 (1.4, 9.5)	3.1 (2.0, 4.4)	ns	0.05	ns
CRP, mg/dl, pre-operation	0.7 (0.5, 1.1)	0.5 (0.1, 0.5)	0.3 (0.1, 0.5)	< 0.05	< 0.01	ns
CRP, mg/dl, POD1	5.3 (2.9, 8.2)	6.1 (3.9, 8.1)	3.5 (2.0, 4.4)	ns	ns	< 0.005

Unless otherwise specified, categorical data are expressed as n (%) and continuous data are expressed as median (25^th^, 75^th ^percentile). CPB, cardiopulmonary bypass; DSC, delayed sternal closure; PCT, procalcitonin; CRP, C-reactive protein; POD, post-operative day.

We next performed multiple linear regression modeling controlling for surgical group, pre-operative AP activity, and pre-operative PCT/CRP levels (Table 5). Based on these models there was no significant association between AP activity and CRP level on POD1. We did, however, find a strong association between PCT level and AP activity on POD1. By our model, each 10 U/L decrease in AP activity on POD1 predicted a 14% increase in PCT level (*P *< 0.01).

## Discussion

The primary aim of this study was to assess the changes in total serum AP activity around cardiothoracic surgery in infants. Our study is the largest to date to examine serum AP activity in any patient population undergoing cardiothoracic surgery. In 1974, Neutze *et al*. briefly described a post-operative decrease in total serum AP activity in 15 children undergoing cardiac surgery with profound hypothermia and limited CPB [19]. The authors did not further explore the etiology or clinical changes associated with decreased AP. Lum *et al*. followed activity in 25 adult CPB patients and showed a 48% decrease in AP activity relative to baseline, compared to a decrease of approximately 15% in non-cardiac surgery patients [18]. Serial AP activity demonstrated a nadir on POD1 with subsequent recovery over the next 7 to 10 days.

In our population, pre-operative AP activity varied significantly between surgical groups. Specifically, the CPB group showed higher pre-operative activity than the non-CPB and DSC groups. These differences were anticipated given the older age of the CPB cohort and the normal age variations in AP activity during infancy [3]. However, it is possible that additional factors such as pre-operative cardiovascular instability and inflammation could modulate pre-operative AP activity. While no patients received enteral feeding within six hours of surgery, enteral feeding status in the days prior to surgery may also have played a role.

Despite these pre-operative differences, we found that AP activity decreased following surgery in almost every case. Often this drop was quite profound. DSC patients showed the greatest relative drop in AP activity with an average decrease > 60%. We also found that these changes were not exclusive to infants exposed to CPB. While the decrease was generally smaller, eight out of nine patients in the non-CPB group exhibited a decrease in their AP activity. The one patient who underwent DSC without CPB exhibited a greater AP decrease of 132 U/L, suggesting that large decreases in AP activity following surgery cannot be purely ascribed to the use of CPB.

Our study was not designed to determine the etiology of decline in AP activity following cardiothoracic surgery. However, we are able to make three preliminary observations. First, removal of AP by the circuit during hemofiltration is an unlikely etiology, as AP is a large molecule (> 65,000 Daltons) and the Sieving coefficient through a polyethersulfone membrane is effectively zero. Second, in contrast to the findings of Lum *et al*. [18], total protein and albumin increased in our patients following surgery, and changes in total protein and albumin did not correlate with changes in AP. These findings make dilution less likely as an etiology, although albumin replacement was not monitored in this study. Third, liver injury is also unlikely as a mechanism, as AP activity was inversely related to ALT, the opposite of the typical pattern in liver congestion or injury [1].

The secondary objective of our study was to examine the relationship between decreased AP activity to requirements and duration of post-operative support, and short term clinical outcomes. In our population, lower total AP activity on POD1 was independently associated with increased intensity and duration of post-operative support, including higher vasoactive and inotropic medication requirements, prolonged intubation time, and increased length of hospital stay. The change in AP activity was not as significant as the absolute activity on POD1, in predicting the need for post-operative support. While the predicted change in support for each unit decrease in AP activity is relatively small, the large differences in post-operative AP activity make these predicted changes clinically relevant. For example, comparing patients at the 25^th ^and 75^th ^percentile in the CPB group (AP 77 vs. 166 U/L), those in the 25^th ^percentile would be expected to have an approximate increase in VIS of 6 (equivalent to the addition of 6 mcg/kg/minute of dopamine or 0.06 mcg/kg/minute of epinephrine), a 50% increase in intubation time, and a 40% increase in length of hospital stay. We did note a small trend towards increased odds of cardiac arrest or death in patients with lower AP activity. In particular, AP activity below 90 U/L appeared to be a sensitive predictor of cardiac arrest or death in our population, and might be a candidate threshold for future observational and interventional research. However, the study size was insufficient to draw any definitive conclusions regarding post-operative AP activity and cardiac arrest or death.

Given these associations with post-operative support and the trend towards differences in the frequency of poor outcomes, AP activity may have some utility as a predictor of increased post-operative support. We did not, however, examine its predictive strength compared to other available biomarkers (such as lactate trends) or scoring systems (such as VIS) already proven to predict differences in outcome. Of greater potential interest, however, is the question of whether AP activity is simply a marker of disease severity, or if AP has a true biologic role in modulating the response to cardiothoracic surgery. Due to the observational design of the study, we cannot directly draw conclusions about cause and effect. Recent literature, however, has suggested that AP may be active in multiple inflammatory and ischemic settings [2-7,10,12-14,16]. AP displays various potentially beneficial actions including modulation of nitric oxide synthase [13] and dephosphorylation of extracellular ATP [23]. The link between AP and inflammation is also a topic of active research. AP is capable of dephosphorylating the lipid-A moiety of endotoxin, converting it to a non-toxic monophosphoryl product [2-7]. AP may also target additional bacterial components such as CpG DNA and flagellin [4]. In cardiac surgery, endotoxin release from the intestine is thought to be one trigger for systemic inflammation, and AP may play a role in reducing this inflammation [8]. To assess if there was a potential link between AP and systemic inflammation in our population, we examined the association between AP and the two inflammatory biomarkers available for analysis through the parent PCT study (PCT and CRP). We were able to demonstrate that lower AP activity on POD1 was strongly associated with increased PCT but not CRP levels. PCT is highly activated by endotoxin [24-26], and prior pediatric studies of cardiac arrest and CPB have shown that elevated levels of PCT, but not CRP, are predictive of poor outcome [24-26]. In this study AP activity was also inversely correlated with PCT levels before the operation, generally in the setting of poor systemic blood flow and lactic acidosis. These data provide some initial evidence for a possible biologic mechanism for AP in our population.

If low AP activity decreases the protective host response to cardiothoracic surgery, then supplementation of AP to higher levels could potentially allow for a reduction in post-operative support requirements. Evidence in favor of AP as a therapeutic agent in various disease processes has recently been established. Enteral administration of AP has been shown to improve outcomes in rat models of necrotizing enterocolitis and Crohn's Disease [10,12]. Administration of Resolvin-E1 in a rat model of Crohn's disease increased expression of intestinal AP and reduced disease activity, improvements which were blunted with inhibition of intestinal AP activity [5]. Administration of exogenous AP has also been effective in animal models of systemic inflammation [6,14].

Several human trials have been performed using AP supplementation. An uncontrolled trial in adults with ulcerative colitis showed that administration of oral AP for seven days resulted in decreased CRP levels and improved clinical response scores [7]. Heemskerk, *et al*. assessed the effects of AP on renal injury in adult patients with severe sepsis/septic shock [13]. AP-treated patients showed an improvement in serum creatinine and glutathione *S*-transferase A1-1 compared to controls, possibly due to a demonstrated reduction in the expression of renal nitric oxide synthase. In a follow-up study, Pickkers, *et al*. showed improvement in a combined renal function outcome as well as a reduction in systemic inflammatory markers in the AP group compared to placebo [23]. In addition to the effects of AP on endotoxin, this group also suggested a role for AP in the dephosphorylation of extracellular ATP. Kats, *et al*. evaluated the effects of exogenous AP on inflammation and outcomes in adult patients undergoing coronary artery bypass grafting [8]. Given the overall favorable clinical course, the study was underpowered to assess a difference in surgical outcomes. However, AP-treated patients did not demonstrate the post-operative rise in the inflammatory markers TNF α, IL-6, and IL-8 seen in a portion of control patients. The authors hypothesized that AP might play a role in preventing post-bypass inflammation, potentially by limiting endotoxin leak from the hypoperfused gastrointestinal tract.

In summary, our study confirms that the decrease in AP activity seen in prior studies reliably occurs in infants undergoing cardiothoracic surgery. Low AP activity was, in turn, associated with the need for increased post-operative support. Patients with low post-operative AP activity may also have an increased risk of cardiac arrest or death, but our study was underpowered to confirm this association. Low post-operative AP activity was significantly associated with elevated PCT levels, providing limited initial evidence for a link between AP and one pathway of systemic inflammation. Based on these results, we believe serum AP may represent an important mediator and potential therapeutic target following cardiothoracic surgery in infants.

There are several limitations to our study. First, it represents a single center experience and generalizability to other centers has not been established. Second, the protocol was designed to answer questions about procalcitonin and CRP rather than AP. The research was therefore restricted by the structure and sample size of the parent study. The sample size was underpowered to assess more rare events such as mortality, cardiac arrest, and the need for mechanical support. Small sample size also limited the scope of covariate assessment in our multivariate analysis. The sub-analysis design limited the collection of additional data of interest, such as specific changes in isoforms, the timing of AP recovery, broader inflammatory biomarker analysis, and endotoxin assays. These aspects should be addressed in future studies. One specific issue of the parent study design concerns the protocols for assessing AP activity. No AP activity was sampled in the very early post-operative period (0 to 6 hours), and data could have been missed on the kinetics, etiology, and relationship to early (POD 0) deaths. POD 1 AP activity was not mandated in the non-CPB group resulting in three patients who could not be analyzed, and potentially limiting the conclusions that can be drawn from this group. Also, all AP activity was assessed at the same time point on POD1 regardless of the time of surgery, potentially introducing an element of bias if surgery performed later in the day differed substantially from morning surgery. Finally, as mentioned previously, this study is observational in design and cannot establish causality. Further research is required to assess whether modulation of AP activity in the peri-operative period will affect post-operative support, inflammation, or outcomes.

## Conclusions

Infants undergoing cardiothoracic surgery experience a predictable post-operative decrease in serum alkaline phosphatase activity. This decrease is more marked in patients undergoing CPB, but is not dependent on the use of bypass. Low alkaline phosphatase activity on post-operative day 1 is independently associated with increased requirement for vasoactive/inotropic medication, prolonged intubation time, and prolonged hospital stay with a trend towards increased odds of cardiac arrest or death. Decreased AP activity is associated with increased PCT levels both before and after surgery.

## Key messages

• AP functions as part of the host defense against inflammation in part through the detoxification of endotoxin and other bacterial products.

• Serum AP activity declines following cardiovascular surgery in infants.

• Low AP activity following cardiothoracic surgery in infants is independently associated with increased vasoactive/inotropic requirements, intubation time, and length of hospital stay.

• Low AP activity is associated with increased PCT levels.

## Abbreviations

AP: alkaline phosphatase; CICU: cardiac intensive care unit; CPB: cardiopulmonary bypass; CRP: C-reactive protein; DHCA: deep hypothermic circulatory arrest; DSC: delayed sternal closure; LFTs: liver function tests; OR: odds ratio; PCT: procalcitonin; PLE: protein losing enteropathy: POD1: post-operative day one; VIS: vasoactive-inotropic score.

## Competing interests

BRAHMS, USA donated samples of the procalcitonin assay to the clinical laboratory at the Children's Hospital, Colorado for clinical or research use. These samples were then designated for use in this study by the clinical laboratory without additional direction from BRAHMS. All authors declare that they have no conflicts of interest.

## Authors' contributions

JD developed the hypothesis in conjunction with JK and EDC. JD, ST, and AH were responsible for the literature search, protocol creation, and enrollment of all patients. Data collection was performed by JD. Data/statistical analyses were performed by ST and JD. JD, ST, AH, DSL, JJ, JK, and EDC wrote/edited the report. All authors contributed to the manuscript and approved the final version of the paper.
